# Predicting Protein–Protein Interactions via Gated Graph Attention Signed Network

**DOI:** 10.3390/biom11060799

**Published:** 2021-05-28

**Authors:** Zhijie Xiang, Weijia Gong, Zehui Li, Xue Yang, Jihua Wang, Hong Wang

**Affiliations:** 1 School of Information Science and Engineering, Shandong Normal University, Jinan 250014, China; 201811010415@stu.sdnu.edu.cn (Z.X.); 201811990134@stu.sdnu.edu.cn (W.G.); 201811010322@stu.sdnu.edu.cn (Z.L.); 2020020935@stu.sdnu.edu.cn (X.Y.); Wangjihua@sdnu.edu.cn (J.W.); 2 Shandong Provincial Key Laboratory for Distributed Computer Software Novel Technology, Shandong Normal University, Jinan 250014, China

**Keywords:** protein–protein interactions (PPIs), PPI signed network, link sign prediction, attention mechanism, gating mechanism

## Abstract

Protein–protein interactions (PPIs) play a key role in signal transduction and pharmacogenomics, and hence, accurate PPI prediction is crucial. Graph structures have received increasing attention owing to their outstanding performance in machine learning. In practice, PPIs can be expressed as a signed network (i.e., graph structure), wherein the nodes in the network represent proteins, and edges represent the interactions (positive or negative effects) of protein nodes. PPI predictions can be realized by predicting the links of the signed network; therefore, the use of gated graph attention for signed networks (SN-GGAT) is proposed herein. First, the concept of graph attention network (GAT) is applied to signed networks, in which “attention” represents the weight of neighbor nodes, and GAT updates the node features through the weighted aggregation of neighbor nodes. Then, the gating mechanism is defined and combined with the balance theory to obtain the high-order relations of protein nodes to improve the attention effect, making the attention mechanism follow the principle of “low-order high attention, high-order low attention, different signs opposite”. PPIs are subsequently predicted on the Saccharomyces cerevisiae core dataset and the Human dataset. The test results demonstrate that the proposed method exhibits strong competitiveness.

## 1. Introduction

Proteins inside cells do not function alone, and they must interact with other proteins to perform their functions. Therefore, studying protein–protein interactions (PPIs) is necessary for understanding various biological processes in cells, such as gene transcription, that involve multiple protein interactions. The accurate prediction of unknown PPIs reveals the function of proteins at the molecular level and is critical for revealing life activity rules, e.g., growth, development, differentiation, and apoptosis. In addition, accurate PPI prediction provides an important theoretical basis for discussing the mechanisms of major diseases, disease treatment, disease prevention, and new drug development.

PPIs control nearly all cellular processes and play an important role in the execution of various physiological functions. Therefore, PPI prediction has been extensively studied, and as such, many methods have been proposed, including biological experimental methods and calculation methods. Currently, the experimental methods for PPI identification mainly include affinity purification mass spectrometry (AP-MS) [1] and yeast two-hybrid system (Y2H) [2]. In recent decades, proteomics based on mass spectrometry (MS) has become an important technique for identifying PPIs. One method of AP-MS is to label the cells of the experimental group and the control group, respectively, by the method of stable isotope labeling with amino acids in cell cultures (SILAC) [3], and then carry out a co-immunoprecipitation (Co-IP) [4] experiment. The immune complex is separated by the specific reaction between the antigen and antibody, and then the protein in the immune complex is detected by liquid chromatography tandem mass spectrometry (LC-MS/MS). When the content of a protein in the experimental group and the control group reaches statistical difference, it can be observed that the protein interacts with the studied protein. This method can greatly reduce the possibility of false positive results of protein–protein interaction. Y2H was initially established by Fields et al. [2] when they studied the properties of yeast transcription factor Gal4. After continuous improvement, Y2H has developed into a mature protein–protein interaction research tool. Y2H is a system in which the two proteins to be studied are cloned into a DNA-binding domain (DNA-BD) and activation domain (AD) of a transcription activator (Gal4, etc.) of yeast expression plasmid, respectively, to construct a fusion expression vector, and then the interaction between the two proteins is analyzed from the expression products. Y2H can also sensitively detect weak and transient interactions between proteins through the expression products of reporter genes. This method is a highly sensitive technology to study the relationship between proteins. In addition, there are many other experimental methods for PPI prediction, such as phage display technology [5] protein chip technology [6], and surface plasmon resonance technology [7]. However, detecting PPIs in biological experiments is inefficient, time-consuming, and laborious; thus, such methods are unsuitable for large-scale PPI detection.

In recent years, structure- and sequence-based PPI prediction calculation methods have been proposed. Structure-based prediction methods are constrained by the experimentally determined protein structure, and sequence-based prediction methods include statistical and machine learning methods. Statistics-based prediction methods primarily include the mirror tree [8] and the co-evolutionary differences (CD) [9]. The mirror tree method is based on observing the correspondence between the phylogenetic trees of related proteins in systems, such as ligands and receptors, and employs a new method to discover possible protein interactions by comparing the evolutionary distances between related protein family sequences. The co-evolutionary differences method does not use multivariate comparison. Thus, it requires less time than other comparison methods. Prediction methods based on machine learning primarily include autocovariance (AC) and support vector machine (SVM) [10], similarity comparison [11], the amino acid composition (AAC) method [12], universal in silico predictor of PPIs (UNISPPI) [13] and the ETB-Viterbi [14]. The advantage of the AC and SVM method is that autocovariance contains the remote interaction information of amino acid residues, which is crucial in PPI identification. The similarity comparison method predicts PPIs according to the pairwise similarity of the primary protein structure, and the amino acid composition method is suitable for any protein sequence, particularly when domain information is lacking. The universal in silico predictor of the PPI method uses a small number of features to train decision tree classifiers. The advantages of this method are low calculation cost and simple implementation. The disadvantage is that decision tree classifiers typically suffer overfitting problems. The ETB-Viterbi method can capture long-distance correlation to improve prediction accuracy and is unaffected by the sequence direction.The calculation methods for predicting PPIs can not only compute large-scale protein interaction data but also have advantages of high accuracy and low cost.

In the real world, many application scenarios can be represented as graphs or networks, wherein nodes represent entities, and edges represent the relationships among entities. Compared with the traditional network that exclusively comprises positive edges, the signed network can express more abundant semantic information and more accurate expression of the actual scene; thus, signed networks are used widely, e.g., in Epinions consumer review networks, Slashdot news review networks, and organizations or groups interaction networks [15]. The edge signs signify the user’s emotional tendency (e.g., like or do not like) regarding a comment. Inspired by this, we express PPIs as a signed network, wherein nodes in the network represent proteins, and connecting edges represent positive or negative interactions between proteins, where positive means the presence and negative means the absence of interaction. The PPI prediction task can be transformed into a link prediction task for the PPI signed network created herein. Research has revealed that the negative edge of a signed network has an important impact on the network structure and node representation [16]. The positive edge renders nodes increasingly similar, whereas the negative edge renders nodes increasingly different. Therefore, in the analysis and study of PPI networks, positive and negative edges must be comprehensively analyzed. However, the structure of a signed network is more complex than that of traditional unsigned networks. In the low-dimensional node representation space of signed networks, the distance between two nodes connected by a positive edge should be less than that of two nodes connected by a negative edge. The traditional representation learning method of unsigned networks cannot be applied directly to signed networks; consequently, researching PPIs based on signed networks is extremely challenging.

Currently, graph neural networks (GNNs) have attracted significant research interest in the field of deep learning, particularly in machine learning tasks, such as link prediction [17,18]. GNNs introduce a neural network into graph data by defining convolution [19] and attention [20]. After the graph convolution network (GCN) was proposed, Velickovic et al. further proposed the use of GAT [20], which utilizes attention coefficients (i.e., weight coefficients) to aggregate the features of neighbor nodes to the central node and uses local stationarity on the graph to learn new node feature expressions. By learning the weight of neighbors, GAT can realize the weighted aggregation of neighbors, rendering GAT more robust to noisy neighbors and giving the model certain interpretability via the attention mechanism [20]. To some extent, GAT is more powerful than GCN because, in the GAT method, the correlation between node features is better integrated into the model. In addition, GAT can not only simultaneously handle multiple nodes of different degrees that are highly parallel, but it also does not rely on graph structure information, thus making it exhibit strong generalizability. Furthermore, the GAT model can be applied to graph-based inductive and transductive learning problems effectively. However, traditional GAT cannot compute a network with negative edges and can only aggregate and update nodes on an unsigned network. A network comprising PPIs is a signed network; therefore, the above limitation of GAT inspired our research on its application to signed networks. In addition, GAT has scope for improvement: first, GAT only considers the influence of low-order neighbors and ignores the influence of high-order neighbors. Second, the balance theory is critical in signed network research; however, it has not been integrated into GAT-based methods. Overall, current GAT-based methods do not have the expected features, i.e., focusing on the sign of the edges, considering high-order neighbor information, and the interpretability of the balance theory.

To overcome the limitation of the inapplicability of GAT to signed networks, the attention mechanism must be improved to effectively predict the unknown links on the signed networks to realize PPI prediction on PPI signed networks. This is the primary focus of the present study.

To solve these problems, the method of gated graph attention for signed networks (SN-GGAT) is proposed herein and subsequently used to predict the links in a PPI network. The primary contributions of this study are summarized as follows:

1. PPIs are transformed into a signed network with rich semantic information, and the proposed SN-GGAT method is applied to the network, thus enabling the accurate prediction of unknown sign information (i.e., interactions) between proteins.
2. We propose the application of GAT to signed networks while retaining the original advantage of GAT and expressing the polarity of the edge more accurately.
3. We define a gating mechanism to determine high-order neighbors that affect a node and innovatively define an attention mechanism to demonstrate that the low-order neighbors of a node have a relatively greater influence on a node. In comparison, high-order neighbors have less influence.
4. To obtain a good interpretative network embedding, we strengthen the constraint of the balance theory on the sign propagation process.

## 2. Materials and Methods

### 2.1. Materials

#### 2.1.1. *Saccharomyces cerevisiae* Core Dataset

One of the PPI datasets used in this study was taken from the Saccharomyces cerevisiae core dataset (version: Scere20170205) in the database of interacting proteins (DIP) [21]. The 5470 protein pairs in the dataset were used as the positive sample set in the test. The *S. cerevisiae* core dataset is available online (https://dip.doe-mbi.ucla.edu/dip/Download.cgi?SM=7&TX=4932, accessed on 10 February 2021).

Since the non-interacting pairs were not readily available, we drew on the strategies in the literature [10,22,23] to construct the negative sample set. Here, we followed the assumption that there is no interaction between proteins at different subcellular localizations in the cell, i.e., we randomly paired proteins with different subcellular location information, and the resulting protein pair was considered non-interactive. The subcellular localization information of proteins can be obtained from the UniProt database [24]. Note that there is a small amount of subcellular localization information of proteins that cannot be queried, and this information was excluded during random matching. The UniProt database is available online (https://www.uniprot.org/, accessed on 10 February 2021). To ensure data balance, we constructed 5470 negative edges for the PPI network by random pairing. In total, the dataset contained 10,940 pairs of samples (50% positive samples and 50% negative samples).

#### 2.1.2. Human Dataset

The second dataset we used is the Human dataset. The HIPPIE database collects human PPIs with experimental annotations [25]. By considering factors such as the number of publications or the type of experimental support, the reliability score of the interaction was scored. Later, Hampe et al. [26] chose 10% of the highest scoring interactions from the HIPPIE dataset to obtain a high-quality human protein subset. In order to obtain fair comparison results, Liu et al. [27] followed the same strategy as DPPIs [28] on the basis of high-quality human protein subset, eliminating the redundancy of the Human dataset, so that no two PPIs are similar at the sequence level. If at least two sequences have more than 40% sequence identity, two PPIs are considered to be similar.

In this paper, we used the Human dataset used by Liu et al. as the second test dataset, the Human dataset is available online (https://zenodo.org/record/3960077/files/Human.zip?download=1, accessed on 31 March 2021).

### 2.2. Related Definition

Here, we describe relevant definitions involved in the proposed SN-GGAT method. First, we describe some concepts of the basic PPI signed network. Then, we define node update rules for PPI signed networks.

#### 2.2.1. PPI Signed Network

A PPI signed network is a graph structure that can be described by G=(V,E), where *V* is the set of all protein nodes in graph *G*, and *E* is the set of edges between any two protein nodes in graph *G*. Here, the edges between protein nodes *i* and *j* are denoted as e(i,j) and e(i,j)∈E, respectively; e(i,j)=1 represents a positive interaction between protein nodes *i* and *j*; e(i,j)=0 represents an unknown interaction between protein nodes *i* and *j*; and e(i,j)=−1 represents a negative interaction between protein nodes *i* and *j*.

**Figure 1:**
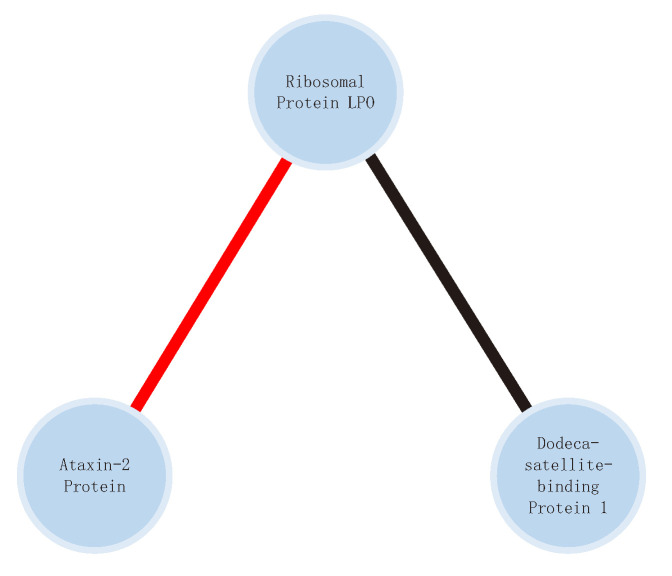
An example of positive and negative interactions between protein nodes. The red edge indicates positive interaction and the black edge indicates negative interaction.

For example, we can learn from the Drosophila melanogaster signed PPI database [29] constructed by Arunachalam V et al. that the interaction between Ribosomal protein LP0 (FlyBase ID: FBgn0000100) and Ataxin-2 protein (FlyBase ID: FBgn0041188) is positive; however, the interaction between Ribosomal protein LP0 and Dodeca-satellite-binding protein 1 (FlyBase ID: FBgn0027835) is negative. Thus, the PPI signed network comprising the Ribosomal protein LP0, Ataxin-2 protein, and Dodeca-satellite-binding protein 1 can be represented as shown in Figure 1. The signed PPI database is available online (https://www.flyrnai.org/SignedPPI/Download.jsp, accessed on 25 January 2021).

(1) $$A\left(i,j\right)=\left\{\begin{array}{cc} -1,\;if\;e(i,j) & =-1\\ 0,\;if\;e(i,j) & =0\\ 1,\;if\;e(i,j) & =1 \end{array}\right.$$

**Figure 2:**
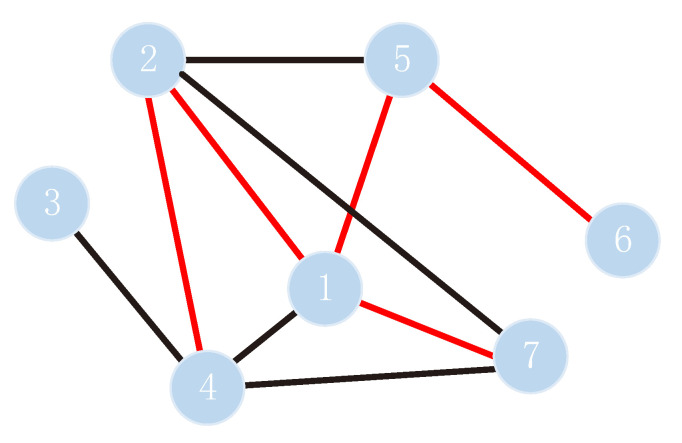
An example of protein–protein interaction (PPI) network. The PPI network consists of seven protein nodes and some connected edges.

To define and explain the adjacency matrix *A* of the PPI signed network more intuitively, we selected seven protein nodes in a PPI network as an example and label these nodes with serial numbers. Simultaneously, we selected part of the edges between these protein nodes to form the PPI signed network (Figure 2). Adjacency matrix *A* is defined in Equation (1), and the adjacency matrix corresponding to Figure 2 is expressed by Equation (2). Here, the link sign prediction task must replace the unknown zeros in the adjacency matrix with the predicted signs.

(2) $$A=\left(\begin{array}{ccccccc} 1 & 1 & 0 & -1 & 1 & 0 & 1\\ 1 & 1 & 0 & 1 & -1 & 0 & -1\\ 0 & 0 & 1 & -1 & 0 & 0 & 0\\ -1 & 1 & -1 & 1 & 0 & 0 & -1\\ 1 & -1 & 0 & 0 & 1 & 1 & 0\\ 0 & 0 & 0 & 0 & 1 & 1 & 0\\ 1 & -1 & 0 & -1 & 0 & 0 & 1 \end{array}\right)$$

#### 2.2.2. Balance Theory

Heider proposed a structural balance model that can use positive and negative links to describe protein interactions or user relationships. The balance theory originally began with analyzing the balance of triangles in signed networks and has since been applied to large-scale link sign prediction. This theory considers all possible combinations of the triplet comprising three nodes, forming four intuitive understandings—a friend of a friend is a friend, an enemy of a friend is an enemy, a friend of an enemy is an enemy, and an enemy of an enemy is a friend.

**Figure 3:**
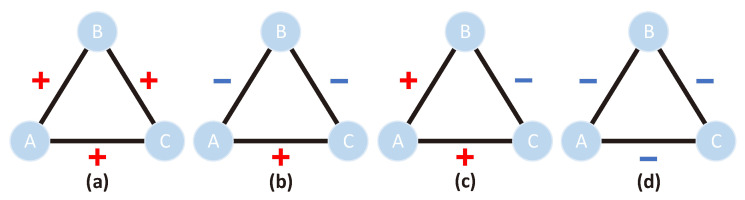
Examples of balanced and unbalanced triangles, where (**a**,**b**) are balanced triangles, and (**c**,**d**) are unbalanced triangles.

According to the structural balance theory, if the product of the signs on the three edges of a triangle is positive, the triangle is structurally balanced; otherwise, it is unbalanced. Here, if we use “+” to represent positive edges, i.e., the positive relationship between two nodes, and “−” to represent the negative edges, i.e., the negative relationship between two nodes, then the structural network of the two triangles in Figure 3a,b is balanced. In contrast, the triangular structural networks in Figure 3c,d are unbalanced. Here, it is worth noting that there are times when the balance theory does not hold in real life, such as G-proteins, which play the role of molecular switches in the process of signal transduction. G-proteins do physically interact with several other proteins, but these proteins do not interact directly. Therefore, this paper states that balance theory has some limitations in specific biological networks. However, in a broad sense, in real life, compared with unbalanced triangles, there are more balanced triangles [30]. In addition, the balance theory has achieved remarkable results in our method. Therefore, we carefully considered balanced triangles in our test.

#### 2.2.3. PPI Signed Accessibility Matrix

If there are *s* paths of length *k* between proteins *i* and *j*, the meaning of length is the number of edges that cross other proteins between two proteins. According to the balance theory, if *p* positive edges and *q* negative edges are obtained, and p+q=s, then the value of the *i*-th row and *j*-th column of the k-order PPI signed accessibility matrix $M_{k}$ is $M_{k}\left(i,j\right)$. This is calculated using Equation (3), where the return value of the sgn(x) function is 1, −1, or 0 when *x* is positive, negative, or zero, respectively.

(3) $$M_{k}\left(i,j\right)=\mathrm{sgn}\left(p-q\right)$$

The 0-order accessibility matrix $M_{0}$ and the first-order accessibility matrix $M_{1}$ are defined first. $M_{0}$ and $M_{1}$ are defined in Equations (4) and (5), respectively.

(4) $$\begin{array}{c} M_{0}=I \end{array}$$

(5) $$\begin{array}{c} M_{1}=A \end{array}$$

$M_{0}$ is equal to the identity matrix, i.e., every node can reach itself in zero steps, and every node is positively related to itself. $M_{1}$ is equal to the adjacency matrix *A* because *A* reflects nodes such that each node can reach itself in a single step, which exactly matches the definition of $M_{1}$.

In addition, we can derive the second-order accessibility matrix $M_{2}$ expression as follows.

(6) $$M_{2}=\mathrm{sgn}\left(M_{1}\cdot M_{1}\right)=\mathrm{sgn}\left(\sum_{k=1}^{n}M_{1}\left(i,k\right)\cdot M_{1}\left(k,j\right)\right)$$

According to Equation (6), the second-order accessibility matrix $M_{2}$ corresponding to Figure 2 can be obtained as follows.

(7) $$M_{2}=\left(\begin{array}{ccccccc} 1 & -1 & 1 & -1 & 1 & 1 & 1\\ -1 & 1 & -1 & 1 & -1 & -1 & -1\\ 1 & -1 & 1 & -1 & 0 & 0 & 1\\ -1 & 1 & -1 & 1 & -1 & 0 & -1\\ 1 & -1 & 0 & -1 & 1 & 1 & 1\\ 1 & -1 & 0 & 0 & 1 & 1 & 0\\ 1 & -1 & 1 & -1 & 1 & 0 & 1 \end{array}\right)$$

Similarly, the third-order accessibility matrix is given in Equation (8). We further derive the expression of the n-order accessibility matrix in Equation (9).

(8) $$\begin{array}{c} M_{3}=\mathrm{sgn}\left(M_{2}\cdot M_{1}\right) \end{array}$$

(9) $$\begin{array}{c} M_{n}=\mathrm{sgn}\left(M_{n-1}\cdot M_{1}\right) \end{array}$$

#### 2.2.4. Node Update Rules for PPI Signed Network

The nodes in a PPI signed network are affected by their m-order neighbors when they update. Using the attention weight, the features of the m-step accessible neighbor nodes are aggregated to the central node to realize node updates. Of these m-order nodes, the attention weight of low-order neighbors is relatively high, and the attention weight symbols of positive and negative links are opposite.

**Figure 4:**
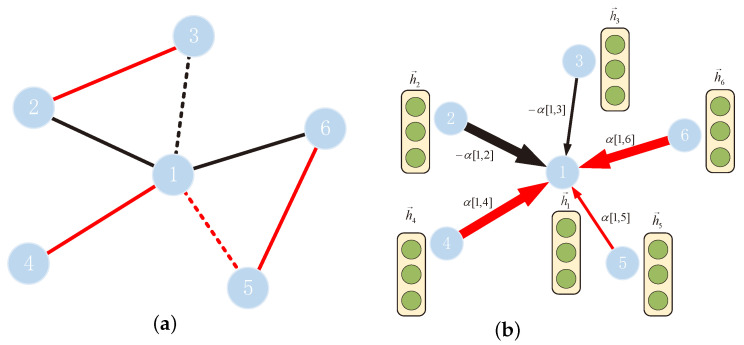
(**a**) PPI signed network, where the solid line represents the first-order neighbor and the dotted line represents the second-order neighbor. (**b**) Update rules for node 1, where the thickness of the arrow represents the relative size of the attention weight.

In the PPI signed network shown in Figure 4a, the solid line represents the first-order neighbor, and the dotted line represents the second-order neighbor. If the value of *m* in the above definition is 2, the update rule of node 1 is shown in Figure 4b. Here, node 1 is affected by its first- and second-order neighbors. The thickness of the arrow represents the relative size of the attention weight. Note that attention α is used to aggregate the neighbor nodes to update node 1.

The specific algorithm and implementation of the attention mechanism and node update are introduced in detail in Section 2.3.

### 2.3. Proposed SN-GGAT Method

Here, we describe the implementation of the proposed SN-GGAT model in detail, including the model structure, gating mechanism, attention mechanism, and algorithm implementation.

#### 2.3.1. Model Framework

**Figure 5:**
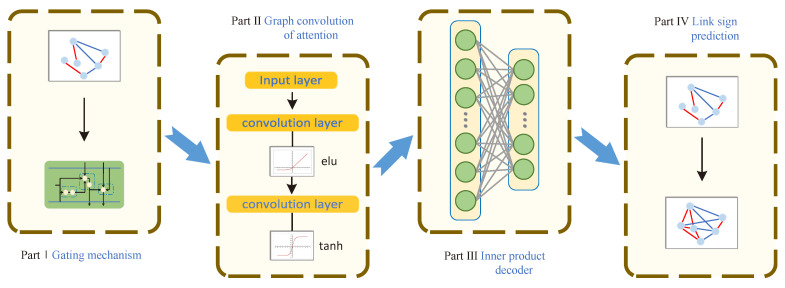
Framework of the proposed SN-GGAT model. Part I uses the gating mechanism to calculate the parameters required by the algorithm; part II executes the algorithm to obtain the feature representation of each node; part III uses the inner product decoder to obtain the reconstructed adjacency matrix; part IV uses the prediction result to verify the accuracy of the model.

The model structure of SN-GGAT is shown in Figure 5, which includes the following four parts.

In Part I, we calculated the adjacency matrix *A*, low-order memory accessibility matrix $M_{m}^{\prime }$, and low-order attention accessibility matrix ${\tilde{M}}_{m}$. These were fed into the gating mechanism, which outputs the corresponding high-order memory accessibility matrix $M_{m+1}^{\prime }$ and high-order attention accessibility matrix ${\tilde{M}}_{m+1}$ as the input for Part II.

In part II, we considered $M_{m+1}^{\prime }$ as the adjacency matrix used by the model. Here, 0 in $M_{m+1}^{\prime }$ represent boundless and a non-zero value represents an edge. The model assigns an attention coefficient to all edges. The role of ${\tilde{M}}_{m+1}$ is to modify and update these attention coefficients such that attention follows the following rules: positive edges are positive, negative edges are negative, low-order neighbor high attention, and high-order neighbor low attention. With these definitions, we employed two convolutional layers to train the node vector’s feature representation. Finally, we added a nonlinear activation function layer, wherein the nonlinear activation function is a hyperbolic tangent function (tanh). We obtained the feature representation of each node through these layers, which forms the input for part III.

In part III, according to the feature representation of nodes, we obtained an adjacency matrix, which is reconstructed. This process is a relatively open problem that can be solved through deep learning. Here, we employed an inner product decoder to calculate node similarity to obtain the reconstructed adjacency matrix, i.e., the link prediction result.

In part IV, model accuracy was verified using the reconstructed adjacency matrix obtained in part III.

#### 2.3.2. Model Interpretation

In the traditional GAT [20] algorithm, convolution is defined as using an attention mechanism to aggregate different neighborhoods differently. The function of the attention mechanism is to assign a weight coefficient to each neighbor node and subsequently updated the central node through convolution summation. Therein, different weights are assigned to different neighbor nodes through GAT, but only the first-order neighbor information of each node is considered. However, there may be other potential edge relationships that have not been mined out in the network. These edge relationships are not considered by GAT, which is inconsistent with the relationships existing in practice because it is not only the first-order neighbor node that affects the node, i.e., a higher-order neighbor node will also have a certain impact on the node or exhibit a certain relationship (positive or negative). For example, X and Y are friends, and their relationship is very good; however, Y and Z have a very poor relationship. According to the balance theory, the relationship between X and Z is very likely to be bad, which means that both Y and Z, i.e., the second-order neighbor of X, have an impact on X. However, Y and Z may have different effects on X, and the impact of first-order neighbors may be even greater, which we discuss in Section 2.3.4.

The algorithm to select high-order neighbor nodes based on the gating mechanism and improving the attention mechanism is employed in the proposed method.

#### 2.3.3. Gating Mechanism

**Figure 6:**
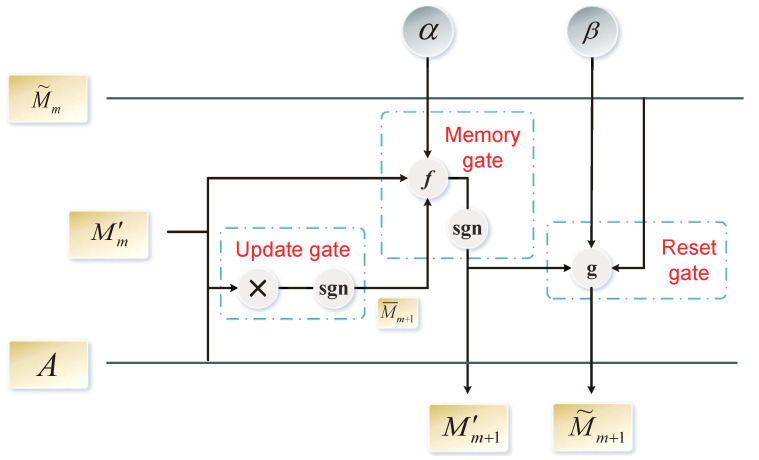
Gating mechanism in the proposed SN-GGAT. It includes update gate, memory gate, and reset gate.

When updating the feature representation of a node, we should consider both the first-order neighbor information of the node and the fact that the high-order neighbor impacts the node. Here, inspired by Long Short-Term Memory (LSTM) [31] and Gate Recurrent Unit (GRU) [32], update, memory, and reset gates are proposed to obtain gating, memory, and attention accessibility matrices, respectively. The gating mechanism employed in the proposed SN-GGAT is shown in Figure 6.

The gating unit’s input is the adjacency matrix *A*, memory accessibility matrix $M_{m}^{\prime }$, and attention accessibility matrix ${\tilde{M}}_{m}$ of the previous gating unit’s output. The output is the memory accessibility matrix $M_{m+1}^{\prime }$ and attention accessibility matrix ${\tilde{M}}_{m+1}$. The first-order memory accessibility matrix $M_{1}^{\prime }$ and first-order attention accessibility matrix ${\tilde{M}}_{1}$ are defined as adjacency matrices in Equations (10a) and (10b), respectively.

(10a) $$M_{1}^{\prime }=A$$

(10b) $${\tilde{M}}_{1}=A$$

The update gate considers the m-order memory accessibility matrix $M_{m}^{\prime }$ and adjacency matrix *A* as input, and outputs the m + 1—order gating accessibility matrix ${\bar{M}}_{m+1}$. The specific calculation method is expressed as follows.

(11) $${\bar{M}}_{m+1}=\mathrm{sgn}\left(M_{m}^{\prime }\cdot A\right)$$

In the update process, the high-order accessibility matrix may forget the low-order neighbor information. For example, in the first-order accessibility matrix (adjacency matrix *A*) and second-order accessibility matrix expressed by Equations (2) and (7) in Section 2.2, the edge from nodes 1 to 2 shown by the second-order accessibility matrix is −1, and the edge from nodes 1 to 2 in the first-order accessibility matrix is +1. Here, the higher-order accessibility matrix forgets the low-order edge information.

To solve this problem, the memory gate memorizes the low-order memory accessibility matrix and displays it in the high-order memory accessibility matrix. In this manner, the high-order memory accessibility matrix can represent the high-order neighbor information of the node and ensure that the low-order neighbor information is retained. The memory gate’s inputs are the m-order memory accessibility matrix $M_{m}^{\prime }$ and *m* + 1—order gating accessibility matrix ${\bar{M}}_{m+1}$. The *m* + 1—order memory accessibility matrix $M_{m+1}^{\prime }$ can then be obtained from the memory gate as follows.

(12) $$M_{m+1}^{\prime }=\mathrm{sgn}\left(\left(1-\alpha \right)\cdot {\bar{M}}_{m+1}+\alpha \cdot M_{m}^{\prime }\right)\;\mathrm{where}\;0.5<\alpha <1$$

In the proposed method, low-order neighbor nodes are considered nodes with a higher influence. Here, the higher the order, the lower the influence of neighbor nodes, and the positive and negative links have opposite attention. The reset gate realizes this function, and its inputs are the m-order attention accessibility matrix ${\tilde{M}}_{m}$ and *m* + 1—order memory accessibility matrix $M_{m+1}^{\prime }$. The *m* + 1—order attention accessibility matrix ${\tilde{M}}_{m+1}$ can then be obtained from the reset gate as follows.

(13) $${\tilde{M}}_{m+1}=\left(1-\beta \right)\cdot {\tilde{M}}_{m}+\beta \cdot M_{m+1}^{\prime }\;\mathrm{where}\;0<\beta \leq 1$$

In the reset gate, the smaller the value of β, the lower the influence of high-order neighbors on nodes. For example, when β=1, the influence of high-order neighbors is the same as that of low-order neighbors.

Therefore, the symbolic operation in Figure 6 represents matrix multiplication, function *f* represents f(x,y)=sgn((1−α)·x+α·y), and function *g* represents g(x,y)=(1−β)·x+β·y, where α and β are hyperparameters.

The high-order memory accessibility and high-order attention accessibility matrices are calculated recursively in the gating mechanism according to the value of m. When using GAT to update nodes, the attention mechanism simultaneously considers both low- and high-order neighbors, and reasonably allocates the attention coefficient according to the order, which makes node updates more appropriate and practical, and the prediction results are more accurate.

#### 2.3.4. Attention Mechanism

We present the following explanations for the definition of the attention mechanism in signed networks.

(1) The first-order neighbor of node *i* is the node directly associated with node *i*; therefore, first-order neighbor nodes have the greatest influence on node *i*.
(2) With increasing order, the influence of high-order neighbors of node *i* on node *i* decreases gradually.
(3) The positive and negative links of node *i* have the opposite influence on node *i*.
(4) The above influences specifically refer to attention and are well implemented in the attention accessibility matrix output by the gating mechanism.

In the proposed method, the expression and updating rules of the attention weight are affected by the attention accessibility matrix ${\tilde{M}}_{m}$, which makes the attention weight follow the rule of “low-order high attention, high-order low attention, different signs attention opposite” in a signed network. The attention weight is calculated as follows.

(14) $$\mathrm{coef}\left[i,j\right]=\frac{\exp\left(\;\mathrm{LeakyReLU}\;\left({\vec{a}}^{T}\left[W{\vec{h}}_{i}∥W{\vec{h}}_{j}\right]\right)\right)}{\sum_{k\in N(i)}\exp\left(\;\mathrm{LeakyReLU}\;\left({\vec{a}}^{T}\left[W{\vec{h}}_{i}∥W{\vec{h}}_{k}\right]\right)\right)}$$

(15) $$\alpha \left[i,j\right]=\frac{\mathrm{coef}\left[i,j\right]\cdot {\tilde{M}}_{m}\left[i,j\right]}{\sum_{k\in N(i)}\mathrm{abs}\left(\mathrm{coef}\left[i,k\right]\cdot {\tilde{M}}_{m}\left[i,k\right]\right)}$$

Here, α[i,j] is the attention weight between nodes *i* and *j*, ·T represents transposition, ∣∣ represents the vector connection operation, parameter *W* is used to realize feature dimension transformation of each node, parameter $\vec{\alpha }$ is used to calculate the attention coefficient of nodes *i* to *j*, function abs() is an operation that provides the absolute value, N(i) is the set of neighbor nodes of node *i*, and the expression of N(i) is given in Equation (16), where n_nodes is the total number of nodes.

(16) $$N\left(i\right)=\left\{n|M_{m}^{\prime }\left[i,n\right]\neq 0,0\leq n<n\_nodes\right\}$$

The updating rules of node features are shown in Equation (17). The low- and high-order neighbors of node *i* constitute set N(i), which impacts node *i*.

(17) $${\vec{h}}_{i}=\tanh\left(\sum_{j\in N(i)}\alpha \left[i,j\right]\cdot W{\vec{h}}_{j}\right)$$

#### 2.3.5. Algorithm

The specific implementation algorithm of the proposed SN-GGAT is given in Algorithm 1.

**Algorithm 1** Gated Graph Attention for Signed Network (SN-GGAT)
**Input:**
 PPIs adjacency matrix **A**; The number of nodes **n**; The order of accessibility matrix **m**; Epochs **E**.
**Output:**
 Node feature matrix **Z**; Reconstructed adjacency matrix $A_{r}$.1: ${\bar{M}}_{1},M_{1}^{\prime },{\tilde{M}}_{1}$←*A*2: **if**
*m*! = 1 **then**3:    **for**
i∈{2,…,m}
**do**4:     ${\bar{M}}_{i}\leftarrow \mathrm{sgn}\left(M_{i-1}^{\prime }\cdot A\right)$5:     $M_{i}^{\prime }\leftarrow \mathrm{sgn}\left(\left(1-\alpha \right){\bar{M}}_{i}+\alpha M_{i-1}^{\prime }\right)$6:     ${\tilde{M}}_{i}\leftarrow \left(1-\beta \right){\tilde{M}}_{i-1}+\beta M_{i}^{\prime }$7:    **end**
**for**8:   **end**
**if**9:   **for** epoch ∈ {1, …, E} **do**10:    **for**
*i* ∈ {0, …, *n* − 1} **do**11:     ***coef***
$\left[i,j\right]\leftarrow \mathrm{softmax}\left(\mathrm{LeakyReLU}\left({\vec{a}}^{T}\left[W{\vec{h}}_{i}∥W{\vec{h}}_{j}\right]\right)\right)$12:     $\alpha [i,j]\leftarrow \mathrm{coef}\left[i,j\right]\cdot {\tilde{M}}_{m}\left[i,j\right]/\sum_{k\in N(i)}abs\left(\mathrm{coef}\left[i,k\right]\cdot {\tilde{M}}_{m}\left[i,k\right]\right)$13:     ${\vec{h}}_{i}\leftarrow \tanh\left(\sum_{j\in N(i)}\alpha \left[i,j\right]\cdot W{\vec{h}}_{j}\right)$14:    **end**
**for**15:    $Z\leftarrow \mathrm{concat}\left({\vec{h}}_{0},{\vec{h}}_{1},\ldots ,{\vec{h}}_{n-1}\right)$16:    $A_{r}\leftarrow \mathrm{sgn}\left(Z\cdot Z^{T}\right)$17:    Update parameters with $A_{r}$18: **end**
**for**

Here, in lines 1 to 8, the m-order gating accessibility matrix, m-order memory accessibility matrix, and m-order attention accessibility matrix are obtained by iterating the gating mechanism, and lines 11 to 13 correspond to the node update process.

From lines 15 to 18, we obtained the node’s feature matrix and used the idea of the inner product decoder to calculate node similarity to obtain the reconstructed adjacency matrix. Here, the loss function uses cross entropy with logits when updating parameters.

## 3. Results and Discussion

### 3.1. Link Sign Prediction of the Saccharomyces Cerevisiae PPI Signed Network

In this section, we used the Saccharomyces cerevisiae core dataset to test the validity of the proposed SN-GGAT, and introduce the computation settings, evaluation criteria, test results, and a discussion of the results.

#### 3.1.1. Computation Settings

In the literature [33], the second-order accessibility matrix was used as the feature representation of nodes, and good link prediction results were obtained in signed networks. Therefore, in our algorithm, the second-order accessibility matrix was input as the feature set of the model.

In this test, we set up two attention convolutional layers. The output dimensions of each layer were 128 and 32, respectively; the multi-head attention mechanism was used in the first layer; and the number of heads was 6. The activation function of the first layer used the elu function, which is used in the original GAT algorithm, and the activation function of the second layer used the tanh function. During training, we used the Adam optimizer with a learning rate of 0.002 to optimize the parameters.

In the gating mechanism, we used the second-order memory accessibility matrix and the second-order attention accessibility matrix with the best test results in the node update process, where the hyperparameters were as follows: α=0.8 and β=0.2. Finally, the node embedded representation of the model output participated in the subsequent link prediction.

#### 3.1.2. Evaluation Criteria

In this test, we used three commonly used machine learning evaluation indexes, i.e., accuracy, precision, and recall, which are defined in Equation (18)–(20), respectively.

(18) $$\begin{array}{c} Accuracy=\frac{TP+TN}{TP+FP+TN+FN} \end{array}$$

(19) $$\begin{array}{c} Precision=\frac{TP}{TP+FP} \end{array}$$

(20) $$\begin{array}{c} Recall=\frac{TP}{TP+FN} \end{array}$$

Here, *TP* denotes true positive (representing the number of samples with positive predictive values and positive labels), *FP* denotes false positive (representing the number of samples with positive predictive values but negative labels), *TN* denotes true negative (representing the number of samples with negative predictive values and negative labels), and *FN* denotes false negative (representing the number of samples with negative predictive values but positive labels).

#### 3.1.3. Test Results

We compared the proposed method with the state-of-the-art PPI prediction methods, including Wong’s method [34], Du’s method [35], DeepFE-PPIs [23], and Song’s method [36]. These methods are summarized as follows:

- Wong’s method: this method is a combination of the Rotating Forest (RF) model and a new feature representation for PPIs detection. In this method, the response matrix (PR) method is used to transform the amino acid sequence into a matrix, and then the texture descriptor based on local phase quantization (LPQ) is used to extract the local phrase information in the matrix.
- Du’s method: This method uses a deep neural network to learn protein representation from common protein descriptors effectively, and extracts useful features of protein pairs by a layer-wise abstraction.
- DEEPFE-PPIs: This method employs a new residue representation called Res2vec, which provides effective input for the downstream deep learning model. PPIs can be accurately inferred when protein structure knowledge is completely unknown.
- Song’s method: In this method, a random projection ensemble classifier (RPEC) is used to identify new PPIs based on the evolutionary information contained in protein amino acid sequences.

**Figure 7:**
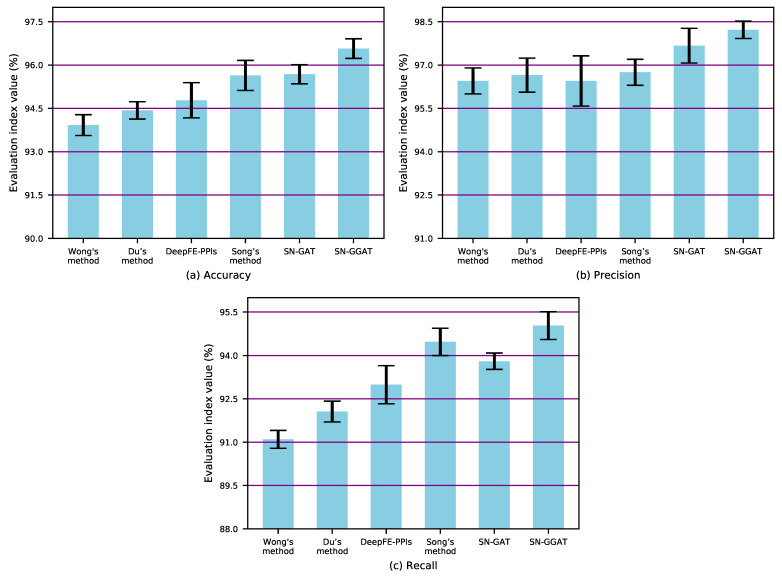
Accuracy, Precision, and Recall values (Mean ± SD) of SN-GGAT method and the compared methods.

We used 5-fold cross validation on the S.cerevisiae core dataset and compared the test results with other state-of-the-art methods. When performing 5-fold cross-validation, we divided the entire dataset into five parts in equal proportions, and took one part as the test set and the other four parts as the training set without repeating each time. In these five tests, each test was run repeatedly three times, and the index values obtained from the 3 times were averaged as the result of each test. The comparison results are shown in Table 1 and Figure 7, where the overall statistical significance level of all tests run is 5% (i.e., *p* < 0.05). Note that SN-GAT is the test result obtained without using the gating mechanism (or the parameter *m* of the gating mechanism is equal to 1), i.e., the memory accessibility and attention accessibility matrices used in the node update process are the adjacency matrix *A* of the PPI signed network.

The results demonstrate that SN-GGAT achieved remarkable PPI prediction results and outperformed the compared methods in all evaluation criteria. Introducing the gating mechanism allows the node to consider high-order neighbor information during the node update process and assigns different weights to both high- and low-order neighbors. The prediction results of SN-GGAT were more accurate than those of SN-GGAT; therefore, the gating mechanism plays an important role in SN-GGAT, which is the reason why our algorithm outperforms other existing prediction algorithms.

In addition, we found a biologically interesting example. In our prediction, there is an interaction between Ctk1 and Snf1. The interaction between the two proteins is not shown in the database, at least not in the Saccharomyces cerevisiae core dataset of DIP database. However, Driessche et al. [37] found a physical interaction between Ctk1 and Snf1 in their two-hybrid system. Ctk1 is a kinase involved in transcriptional control, and Snf1 is a kinase that regulates glucose-dependent genes. Driessche et al. showed that Ctk1 and Snf1 co-regulate GSY2 in vivo by Northern blot analysis. This finding supports the view that Ctk1 interacts with Snf1 in the functional module of cell response to glucose restriction.

#### 3.1.4. Parameter Discussion

**Figure 8:**
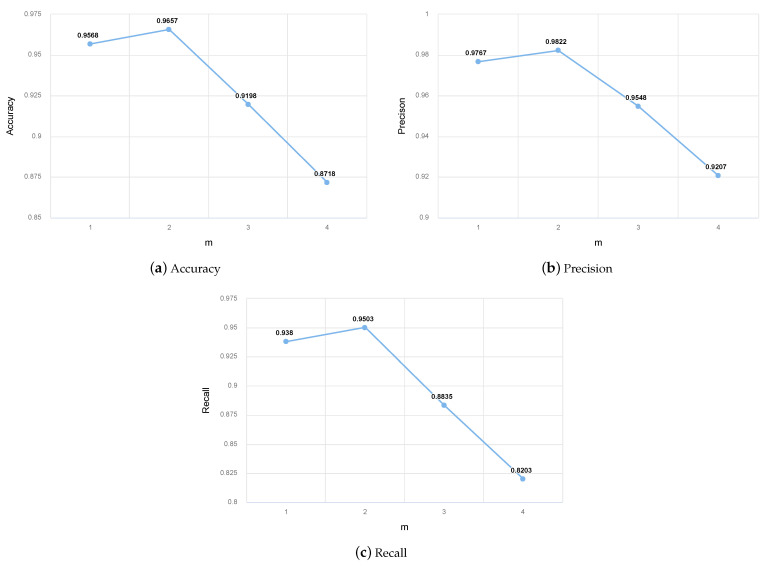
Test results obtained with different *m* values on the S.cerevisiae core dataset. Where (**a**–**c**) are the Accuracy, Precision, and Recall with different *m* values, respectively.

In our algorithm, the most important hyperparameter is parameter *m* of the gating mechanism (the order *m* of the accessibility matrix). After conducting nearly twenty tests, we found that when m=2, the result obtained using the second-order memory accessibility matrix and second-order attention accessibility matrix was excellent. The test results obtained by considering different values of *m* are shown in Figure 8a–c.

As can be observed in the results described above, the index values obtained using the second-order memory accessibility matrix are greater than those obtained using the adjacency matrix (first-order memory accessibility matrix), and model performance was optimal when using the second-order memory accessibility matrix. However, the model’s performance was poor when the third- or fourth-order memory accessibility matrices were used. We summarize possible reasons for these results as follows.

1. The first-order neighbors of a node have the highest influence on the node; therefore, the result obtained using m=1 were second only to the result obtained using m=2.
2. As m=1 only considers first-order neighbor nodes as neighbors and does not consider second-order neighbor nodes, m=2 will consider high-order information more comprehensively. The gating mechanism reasonably allocates the attention weight for the first- and second-order nodes, thus improving the test results when m=2.
3. When m>2, the model’s result was very poor. There may be two reasons for this. First, according to the principle of the defined gating mechanism, the higher the accessibility matrix’s order, the higher the number of high-order nodes that are assigned attention. When the attention weight is calculated using Equation (15), under the influence of high-order neighbors, the proportion of the influence of first-order neighbors on nodes decreases, thereby worsening the model’s final effect. Second, we explain this phenomenon through an intuitive example: my friend (first-order neighbor) has a great influence on me, and my friend’s friend (second-order neighbor) also has a certain influence on me; however, a friend of my friend’s friend (third-order neighbors) may have a minimal influence on me, and I may not even meet them (third-order neighbors) in real life. Therefore, the test results obtained with m=3 are not ideal.

Generally, when m=2, our gating mechanism achieved highly satisfactory results. The subject of considering high-order information without reducing the attention of low-order neighbors to themselves will be researched in future work.

### 3.2. Link Sign Prediction of the Human PPI Signed Network

In this section, we predict the human–protein interaction, verify the performance of our method by comparing it with three state-of-the-art methods, and discuss the biological meanings.

#### 3.2.1. Test Results

**Figure 9:**
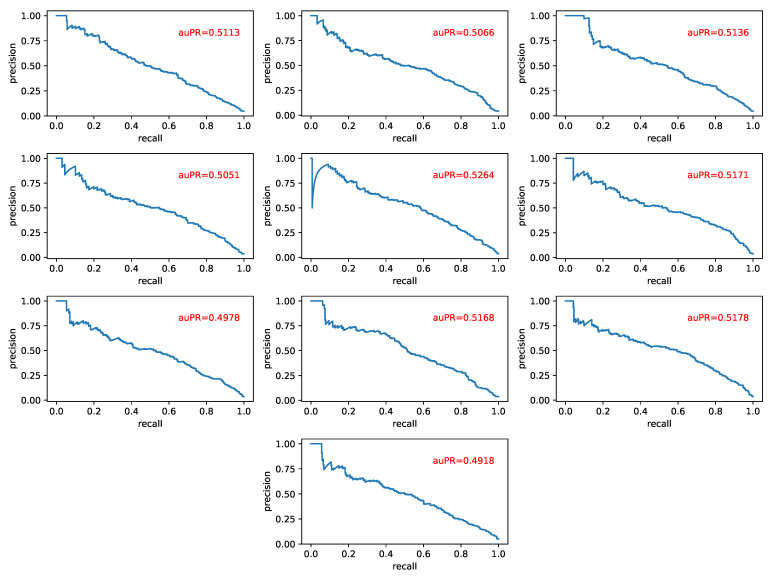
The test results obtained by using the SN-GGAT method to execute the link signed prediction task on the Human dataset, where each sub-graph represents one prediction result.

We predicted the link of the signed network composed of human protein and evaluated the model by using the value of auPR, where auPR refers to the area under the PR (precision-recall) curve. The higher the value of auPR, the better the performance of the model. We performed 10-fold cross validation on the Human dataset, and the test results are shown in Figure 9.

**Figure 10:**
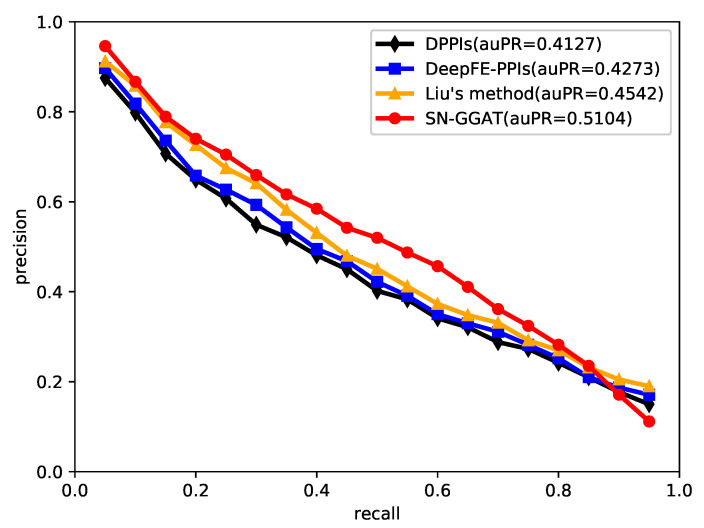
Performance comparison of SN-GGAT with other state-of-the-art methods on the Human dataset. The auPR is the mean of 10-fold cross validation.

We took the average value of ten results, drew the average curve of PR, and compared it with DPPIs [28], DeepFE-PPIs [23], and Liu’s method [27]. The comparison results are shown in Figure 10. DPPIs uses a convolutional neural network combined with random projection and data expansion to predict PPIs, and its auPR value is 0.4127. DeepFE-PPIs employ a new residue representation called Res2vec, which provides effective input for the downstream deep learning model. The auPR value of this method is 0.4273. Liu’s method uses GCNs to learn the location information of proteins in a PPI network and combines the sequence and location information of amino acids to generate strong protein characterization, with an auPR value of 0.4542. The SN-GGAT method has the best performance, and the auPR value is 0.5104, which is higher than the other three methods.

**Figure 11:**
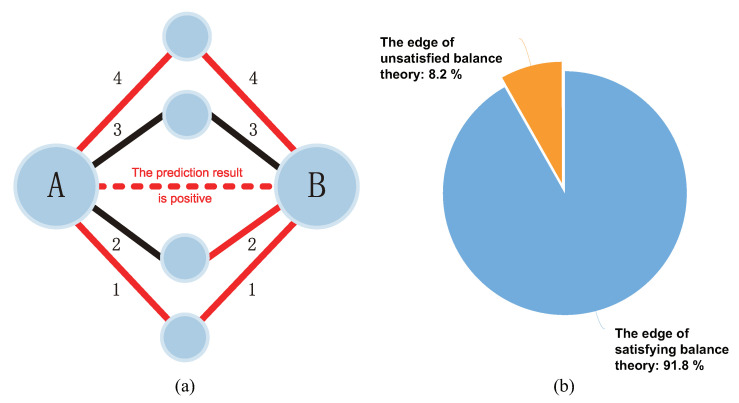
(**a**) An example of PPI satisfying balance theory in signed networks. According to the balance theory, it can be inferred that the connection between protein A and protein B is positive, negative, positive, and positive through routes 1, 2, 3, and 4, respectively. Since the theoretically positive quantity is more than the theoretically negative quantity, if the connection edge between protein A and protein B is predicted to be positive, it means that the predicted protein interaction satisfies the balance theory in the entire signed network. (**b**) The analysis result of the interaction network obtained after the prediction. It shows the proportion of edges satisfying balance theory and unsatisfied balance theory in the entire signed network.

By analyzing the prediction results, we found that most of the predicted interactions satisfy the balance theory in the entire signed network. The explanation of satisfying the balance theory is shown in Figure 11a, and the analysis result is shown in Figure 11b.

This analysis result shows that in the entire signed network after prediction, 91.8% of the connected edges satisfy the balance theory. At the same time, it also shows that the balance theory plays a key role in the gating mechanism and sign propagation process. The introduction of balance theory into SN-GGAT has an important impact on the performance of the model.

#### 3.2.2. Discussion in Biological Meanings

In our test, we constructed the protein–protein interactions as a signed network. The SN-GGAT method expands the PPI network by predicting unknown protein interactions (i.e., the connection edges in the PPI network were significantly increased) so that the entire PPI network is rich in more interactive information. From a biological point of view, SN-GGAT can provide important clues for the in-depth study of protein functions by accurately predicting protein interactions. In the current bioinformatics research, some new methods [38,39,40] using the PPI network to predict protein function have been proposed. SN-GGAT can expand the existing PPI network and increase the training samples of protein function prediction by increasing the number of edges in the protein–protein interaction network, so as to improve the accuracy of protein function prediction. In addition, the accurate prediction of protein interactions can also promote the in-depth study of gene expression regulatory networks in biological life activities and other complex life activities. In sum, the SN-GGAT method we proposed has made considerable contributions to biological research, especially in research based on PPI networks and other bioinformatics-related research.

## 4. Conclusions

Protein–protein interaction usually refers to the binding or chemical reaction between proteins through spatial conformation or chemical bond, while a protein–protein interaction network is composed of proteins through their interactions. These interactions are involved in cell cycle regulation, gene expression regulation, biological signal transduction, and energy and substance metabolism processes. The accurate prediction of protein–protein interactions in biological systems plays an important role in understanding the working principle of proteins, the functional connections between proteins, and the reaction mechanism of biological signals and energy metabolism.

In order to accurately predict PPIs, we characterized PPIs as a signed network and realized PPI prediction via link prediction of the signed network. We developed the SN-GGAT method, which combines the concepts of signed network, balance theory, and accessibility matrix. The proposed method selects high-order neighbor nodes based on a gating mechanism and improves the attention mechanism of the original GAT. In addition, node features are updated according to the rules of “low-order high attention, high-order low attention, different sign attention opposite”; thus, the concept of GAT is extended to signed networks and applied to PPI prediction. We compared the proposed SN-GGAT to four state-of-the-art methods on the Saccharomyces cerevisiae core dataset. The test results demonstrated that the proposed method obtained the highest accuracy and has strong competitiveness. Finally, we tested the proposed method again on the Human protein interaction dataset. We learned that most of the predicted PPIs satisfy the balance theory in the entire signed network. This conclusion shows that the idea of incorporating balance theory into the algorithm is correct and necessary.

In the future, we plan to further study the attention mechanism of signed networks to effectively mine and use hidden high-order information to improve the accuracy of PPI prediction. In addition, the PPI is predicted in this paper, and we will continue in-depth study in the future, such as experimental verification of the predicted results.

## Figures and Tables

**Table 1:** Performance comparison of SN-GGAT with other state-of-the-art methods on the *S. cerevisiae* core dataset.

Method	Test Set	Accuracy (%)	Precision (%)	Recall (%)
SN-GGAT	1	96.43	98.01	94.96
	2	97.04	98.40	95.78
	3	96.25	97.82	94.78
	4	96.34	98.28	94.50
	5	96.81	98.57	95.14
	Average	96.57 ± 0.34	98.22 ± 0.30	95.03 ± 0.48
SN-GAT	1	95.72	97.61	93.95
	2	96.03	97.99	94.14
	3	95.19	96.69	93.85
	4	95.54	97.79	93.40
	5	95.91	98.26	93.67
	Average	95.68 ± 0.33	97.67 ± 0.60	93.80 ± 0.28
Song’s method	Average	95.64 ± 0.52	96.75 ± 0.45	94.47 ± 0.47
DeepFE-PPIs	Average	94.78 ± 0.61	96.45 ± 0.87	92.99 ± 0.66
Du’s method	Average	94.43 ± 0.30	96.65 ± 0.59	92.06 ± 0.36
Wong’s method	Average	93.92 ± 0.36	96.45 ± 0.45	91.10 ± 0.31

## Data Availability

The Saccharomyces cerevisiae core dataset was obtained from the database of interacting proteins (DIP) and are available at https://dip.doe-mbi.ucla.edu/dip/Download.cgi?SM=7&TX=4932 (accessed on 10 February 2021) with the permission of DIP. The UniProt database was obtained from UniProt and is available at https://www.uniprot.org/ (accessed on 10 February 2021) with the permission of UniProt. The Human PPI dataset was obtained from Liu’s site and is available at https://zenodo.org/record/3960077/files/Human.zip?download=1 (accessed on 31 March 2021) with the permission of Liu.

## Abbreviations

The following abbreviations are used in this manuscript:

PPIs	Protein–protein interactions
SN-GGAT	Gated Graph Attention for Signed Networks
GAT	Graph Attention Network
GNNs	Graph Neural Networks
GCN	Graph Convolution Network
